# New species and new records of Tullbergiidae (Arthropoda, Hexapoda, Collembola, Poduromorpha) from Xizang, Southwest China, with genetic distance analysis within the family

**DOI:** 10.3897/zookeys.1286.196797

**Published:** 2026-07-23

**Authors:** Yun Bu, Yan Gao

**Affiliations:** 1 Shanghai Natural History Museum, Shanghai Science & Technology Museum, Shanghai 200041, China Shanghai Natural History Museum, Shanghai Science & Technology Museum Shanghai China

**Keywords:** Chaetotaxy, DNA barcodes, identification key, *
Mesaphorura
*, *
Metaphorura
*, taxonomy

## Abstract

The Tullbergiidae of Xizang, Southwest China, are studied based on specimens newly collected in 2024. *Metaphorura alticola***sp. nov**., which was discovered in two localities at extremely high altitudes of 4000–5300 m in Shannan City, is described and illustrated. It can be distinguished from the similar *M. motuoensis* Bu & Gao, 2017 by the dorsal chaetotaxy, number of vesicles on PAO, and the shape of anal spines on Abd. VI, as well as the large genetic distance of the COI gene. *Mesaphorura macrochaeta* Rusek, 1976, found from several localities in Xizang, is recorded in China for the first time, and Chinese specimens are described and illustrated. In addition, the distributions for *M. hylophila* Rusek, 1982, *M. krausbaueri* (Börner, 1901), *M. pacifica* Rusek, 1976, and *M. yosii* (Rusek, 1967) in Xizang are updated according to the newly collected material. DNA barcodes of mitochondrial COI gene for *Metaphorura alticola***sp. nov**., *M. macrochaeta*, and *M. hylophila* are newly sequenced and analyzed together with 26 sequences of the family extracted from GenBank. The genetic distances of different levels in the family Tullbergiidae are analysed. A key for all tullbergiid species recorded in Xizang and a map of their distributions are also provided.

## Introduction

The family Tullbergiidae Bagnall, 1935 is a group of tiny collembolans with about 230 known species in the world (Bellinger et al. 1996–2024). Fifteen species belonging to nine genera have been recorded in China (Gao and Bu 2022), but only five species have been reported from Xizang (Bu and Gao 2017b, 2019): *Metaphorura motuoensis* Bu & Gao, 2017, *Metaphorura zhongi* Bu & Gao, 2019, *Mesaphorura yosii* (Rusek, 1967), *Mesaphorura hylophila* Rusek, 1982, and *Prabhergia imadatei* Tamura & Zhao, 1996 (in Tamura and Zhao 1996). A comprehensive investigation of soil fauna was conducted in 2024 by Yun Bu during his one-year aid work in Xizang, and plenty of specimens of Tullbergiidae were obtained. After careful study, those materials were identified as six species: one new species of the genus *Metaphorura* Bagnall, 1936, and five known species of the genus *Mesaphorura* Börner, 1901. The new species is illustrated and described in the present paper, and *Mesaphorura macrochaeta* Rusek, 1976, which is recorded from China for the first time, is described based on Chinese specimens. To clarify the genetic divergence, the DNA barcodes of the new species, as well as of *M. macrochaeta* and *M. hylophila* were sequenced and compared. Using all currently available and accessible molecular data of Tullbergiidae, the genetic distances within the family at both the species and genus levels were also analysed.

## Materials and methods

### Sample collection

**Figure 1.**
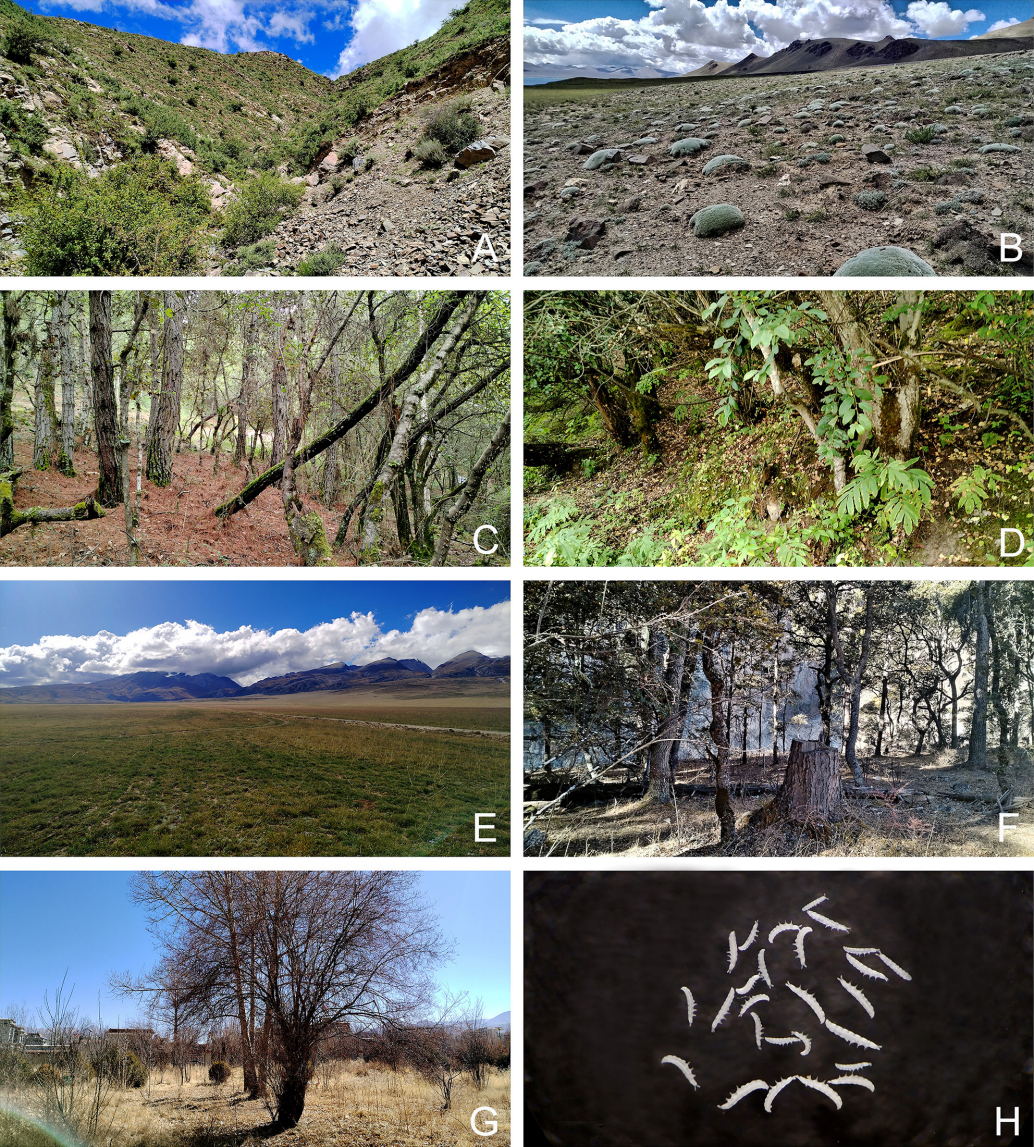
Habitats of collection sites in Xizang and specimens of *Metaphorura alticola* sp. nov. in alcohol. **A**. Sparse scrubland close to Yamdrok Lake, Langkazi County, Shannan City; **B**. Alpine meadow beside the lake Pumoyum Co, Langkazi county, Shannan City; **C**. Broad-leaf forest of Biri Sacred Mountain, Bayi District, Linzhi City; **D**. Bush forest beside lake Basong Co, Gongbujiangda County, Linzhi City; **E**. Grassland in Bange County, Naqu City; **F**. Coniferous forest in Jiali County, Naqu City; **G**. Poplar forest in Chengguan District, Lhasa City; **H**. Specimens of *Metaphorura alticola* sp. nov. in alcohol.

All materials were extracted from soil samples collected in bush, forests, or grasslands of several localities by using Berlese-Tullgren funnels. Specimens were kept in absolute ethanol and frozen. Their habitats are shown in Fig. 1A–G. The map showing the collection sites in Xizang was generated by the software QGIS v. 4.0.1. The basemap layer was retrieved from tianditu (https://tianditu.gov.cn) hosted by the National Platform for Common GeoSpatial Information Services and used to label all sites.

### Morphological study

Specimens were mounted on slides in Hoyer’s solution and dried in an oven at 50 °C for identification. Morphological observations were performed under a phase-contrast microscope Leica DM 2500. Photographs were taken with a Leica DMC 4500 digital camera mounted on the microscope. Line drawings were made using a drawing tube and vectorised with SAI v. 2.0 software. Measurements were made by using an ocular micrometer installed on the microscope. The type specimens are deposited in the Shanghai Natural History Museum (**SNHM**), Shanghai, China. In the description, we used the nomenclature for morphological features following Dunger and Schlitt (2011). Pseudocellar types after Weiner and Najt (1991). Antennal chaetae notation after Rusek (1971). Formula of tibiotarsal chaetotaxy after Fjellberg (1991).

### Abbreviations used in the descriptions

**Th**. thoracic segment,

**Abd**. abdominal segment,

**Ant**. antennal segment,

**Asp**. anal spine,

**PAO** postantennal organ,

**a** anterior setae,

**m** medial setae,

**p** posterior setae,

**pl** pleural setae,

**pso** pseudocelli,

**s** sensillum.

### Molecular experiments

The specimens used for molecular analyses were preserved in absolute ethanol at −20 °C for DNA extraction. For DNA barcodes, total genomic DNA of the entire individual was extracted from one specimen with the Promega genomic DNA purification kit following the manufacturer’s instructions. The primer pair LCO (5’-GGTCAACAAATCATAAAGATATTGG-3’) and HCO (5’-TAAACTTCAGGGTGACCAAAAAATCA-3’) (Folmer et al. 1994) was used for amplification and sequencing.

### Genetic divergence analysis

To analyse the genetic divergences between the new species and other known species, as well as the divergences at different taxonomic levels in Tullbergiidae, four COI DNA barcodes from three species were newly sequenced, and 26 COI gene sequences of 14 Tullbergiidae species and two outgroups of the family Onychiuridae were downloaded from GenBank for use in the analysis. Sequences were aligned and trimmed using BioEdit v. 7.0 (Hall 1999). The detailed information and accession numbers of all sequences analysed in this study are listed in Table 1. A neighbour-joining tree was constructed based on COI gene sequences by MEGA X (Kumar et al. 2018) with the Jukes–Cantor model (Jukes and Cantor 1969) and 1000 bootstrap replicates. The genetic distance (K2P distance) was calculated using MEGA X (Kimura 1980; Kumar et al. 2018).

**Table 1.** Taxonomic information, collection site, size, and GenBank accession number of the partial or complete sequences of COI of the species used in the analysis.

**Species and voucher**	**Genus**	**Location**	**Length (bp)**	**GenBank number**	**References**
*Metaphorura alticola* sp. nov. BY-XZ2024011	* Metaphorura *	China: Xizang	658	PZ254683	**Present study**
*Metaphorura motuoensis* TU2	* Metaphorura *	China: Xizang	1579	OP235283	Li et al. 2022
*Metaphorura affinis* 4G1b1_JC365	* Metaphorura *	Germany	681	KY231093	Chen et al. unpublished
*Metaphorura denisi* Tu2	* Metaphorura *	France: Corsica	658	HQ732072	Greenslade et al. 2011
*Mesaphorura macrochaeta* BY-XZ2024001	* Mesaphorura *	China: Xizang	658	PZ254686	**Present study**
*Mesaphorura macrochaeta* SBS-183	* Mesaphorura *	Denmark	658	MN525426	Krogh unpublished
*Mesaphorura hylophila* BY-XZ2024008	* Mesaphorura *	China: Xizang	658	PZ254684	**Present study**
*Mesaphorura hylophila* BY-XZ2024009	* Mesaphorura *	China: Xizang	658	PZ254685	**Present study**
*Mesaphorura hylophila* AK41b	* Mesaphorura *	Germany	657	PP866837	Merges unpublished
*Mesaphorura krausbaueri* AK42c	* Mesaphorura *	Germany	657	PP866834	Merges unpublished
*Mesaphorura yosii* TU4	* Mesaphorura *	China: Jiangsu	1531	OP235284	Li et al. 2022
*Mesaphorura yosii*	* Mesaphorura *	China: Zhejiang	658	KT799636	Ma et al. unpublished
*Mesaphorura yosii* JPV-68_Dec_ARM3_400_Mesyos_5	* Mesaphorura *	Panama	658	MT357564	Basset et al. 2020
*Mesaphorura yosii* JPV-68_Dec_ARM3_400_Mesyos_4	* Mesaphorura *	Panama	630	MT357566	Basset et al. 2020
*Paratullbergia changfengensis* TU5	* Paratullbergia *	China: Shanghai	1531	OP235285	Li et al. 2022
*Paratullbergia callipygos* c549 fra200814l	* Paratullbergia *	France: Manche	658	PV883181	Stevens and D’Haese 2025
*Stenaphorura denisi* 1F1b1 JC476	* Stenaphorura *	Germany	681	KY231135	Chen et al. unpublished
*Stenaphorura quadrispina* AK39b	* Stenaphorura *	Germany	657	PP866838	Merges unpublished
*Tullbergia bisetosa*	* Tullbergia *	Antarctica: Prince Edward Islands	1527	MK520870	Jagatap et al. 2019
*Tullbergia bisetosa*	* Tullbergia *	Chile: Tierra del Fuego	657	KP027077	Velasco Castrillon unpublished
*Tullbergia bisetosa* Tu6.1	* Tullbergia *	South Africa: Marion Island	606	DQ147412	Myburgh et al. 2007
*Tullbergia bisetosa* Tu6.2	* Tullbergia *	South Africa: Marion Island	606	DQ147413	Myburgh et al. 2007
*Tullbergia bisetosa* Tu6.3	* Tullbergia *	South Africa: Marion Island	606	DQ147414	Myburgh et al. 2007
*Tullbergia bisetosa* Tu6.4	* Tullbergia *	South Africa: Marion Island	606	DQ147415	Myburgh et al. 2007
*Tullbergia mediantarctica* ANTSP1071	* Tullbergia *	Antarctica: Ross Sea	658	MN619502	Collins and Hogg unpublished
*Tullbergia mediantarctica* ANTSP1072	* Tullbergia *	Antarctica: Ross Sea	658	MN619521	Collins and Hogg unpublished
*Tullbergia mediantarctica* ANTSP1073	* Tullbergia *	Antarctica: Ross Sea	658	MN619529	Collins and Hogg unpublished
*Tullbergia mediantarctica* ANTSP1074	* Tullbergia *	Antarctica: Ross Sea	658	MN619479	Collins and Hogg unpublished
*Tullbergia mediantarctica* ANTSP1075	* Tullbergia *	Antarctica: Ross Sea	658	MN619566	Collins and Hogg unpublished
*Tullbergia mixta*	* Tullbergia *	Antarctica: Harmony Point Nelson	1527	MW238520	Cucini et al. 2021
*Onychiurus orientalis*	* Onychiurus *	China: Shanghai	1531	NC006074	Cook et al. 2005
*Thalassaphorura encarpata*	* Thalassaphorura *	China: Jiangsu	1534	MK450463	Sun et al. unpublished

## Results

### Taxonomy

**Family Tullbergiidae Bagnall, 1935**

#### Genus Metaphorura

Taxon classificationAnimaliaPoduromorphaTullbergiidae

Bagnall, 1936 sensu Stach, 1954

B0060C7F-764C-5F58-9C71-B4E8B079E2B0

##### Type species.

*Tullbergia affinis* Börner, 1903.

#### Metaphorura alticola sp. nov.

Taxon classificationAnimaliaPoduromorphaTullbergiidae

10D66DC6-84F0-56A1-A44E-E8E31AE730B4

https://zoobank.org/DBDAFA89-FD76-4319-9E77-12D04A2F836B

**Figure 2.**
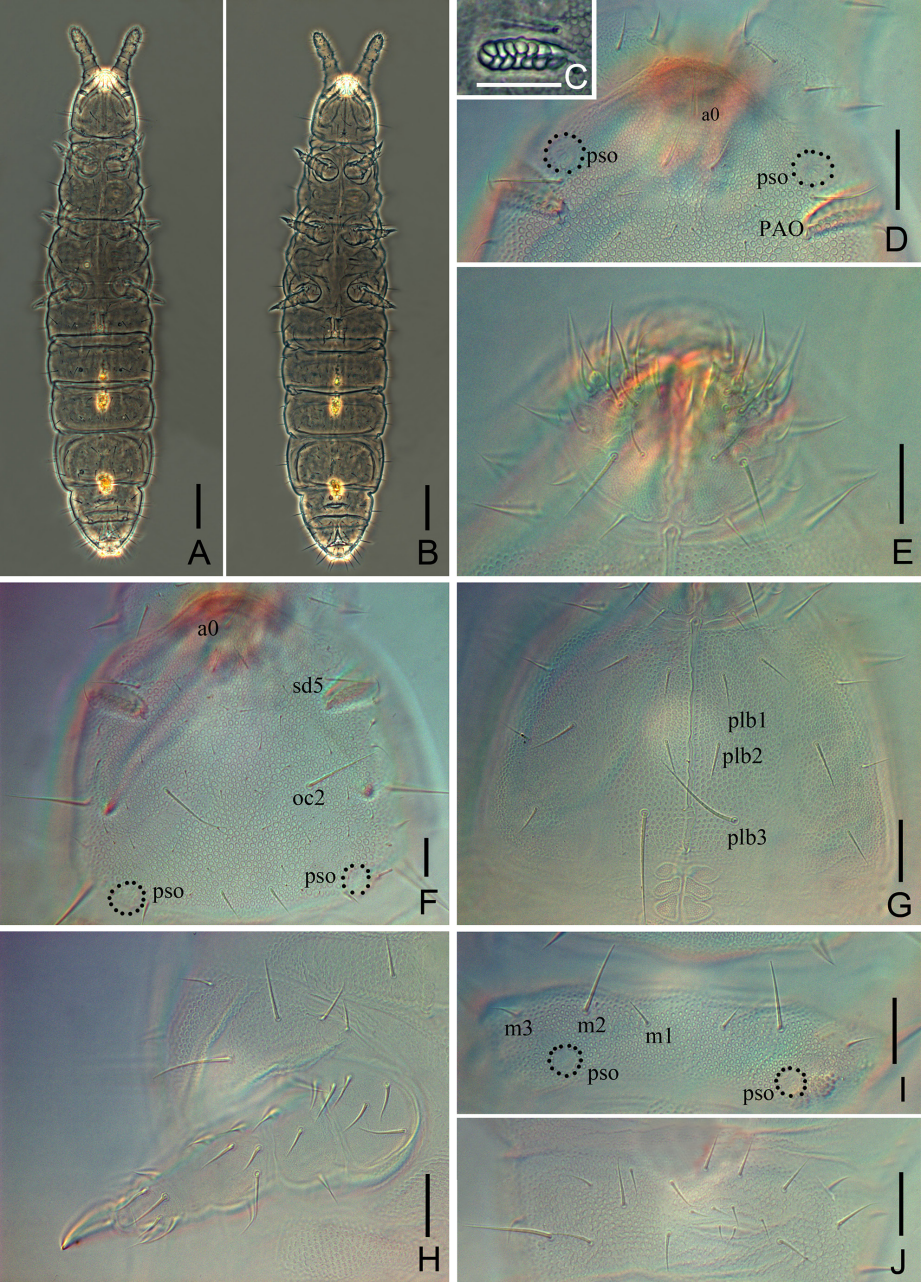
*Metaphorura alticola* sp. nov. **A**. Habitus, dorsal view; **B**. Habitus, ventral view; **C**. Postantennal organ; **D**. Head, anterior part; **E**. Labium; **F**. Head, dorsal view; **G**. Head, ventral view, plb1, 2, 3–postlabial setae; **H**. Leg III; **I**. Th. I; **J**. Female genital plate. Scale bars: 100 μm (**A, B**); 20 μm (**C–J**).

**Figure 3.**
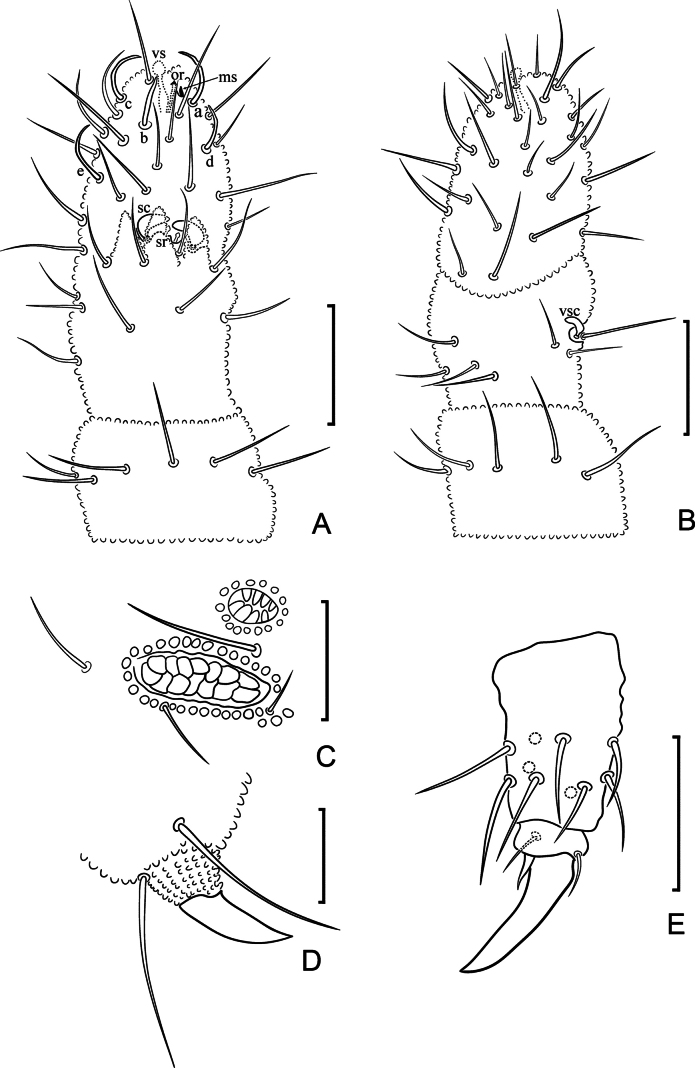
*Metaphorura alticola* sp. nov. **A**. Ant. II–IV, dorsal view, a, b, c, d, e–large sensilla, ms–microsensillum, or–subapical organite, vs– apical vesicles, sc–sensory clubs, sr–sensory rods; **B**. Ant. II–IV, ventral view, vsc–ventral sensory club; **C**. Postantennal organ and pso at base of antenna; **D**. Anal spine, lateral view; **E**. Tibiotarsus III and claw. Scale bars: 20 μm.

**Figure 4.**
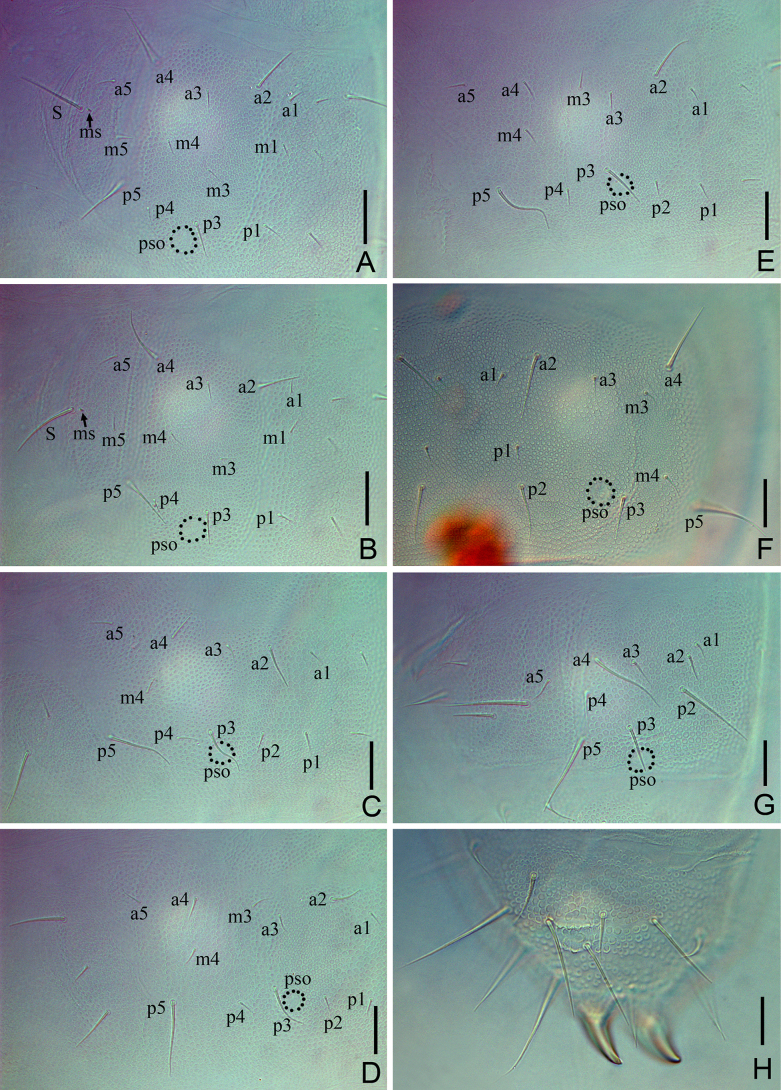
*Metaphorura alticola* sp. nov. **A**. Th. II, left side; **B**. Th. III, left side; **C**. Abd. I, left side; **D**. Abd. II, left side; **E**. Abd. III, left side; **F**. Abd. IV, right side; **G**. Abd. V, left side; **H**. Abd. VI, dorsal view. Scale bars: 20 μm.

**Figure 5.**
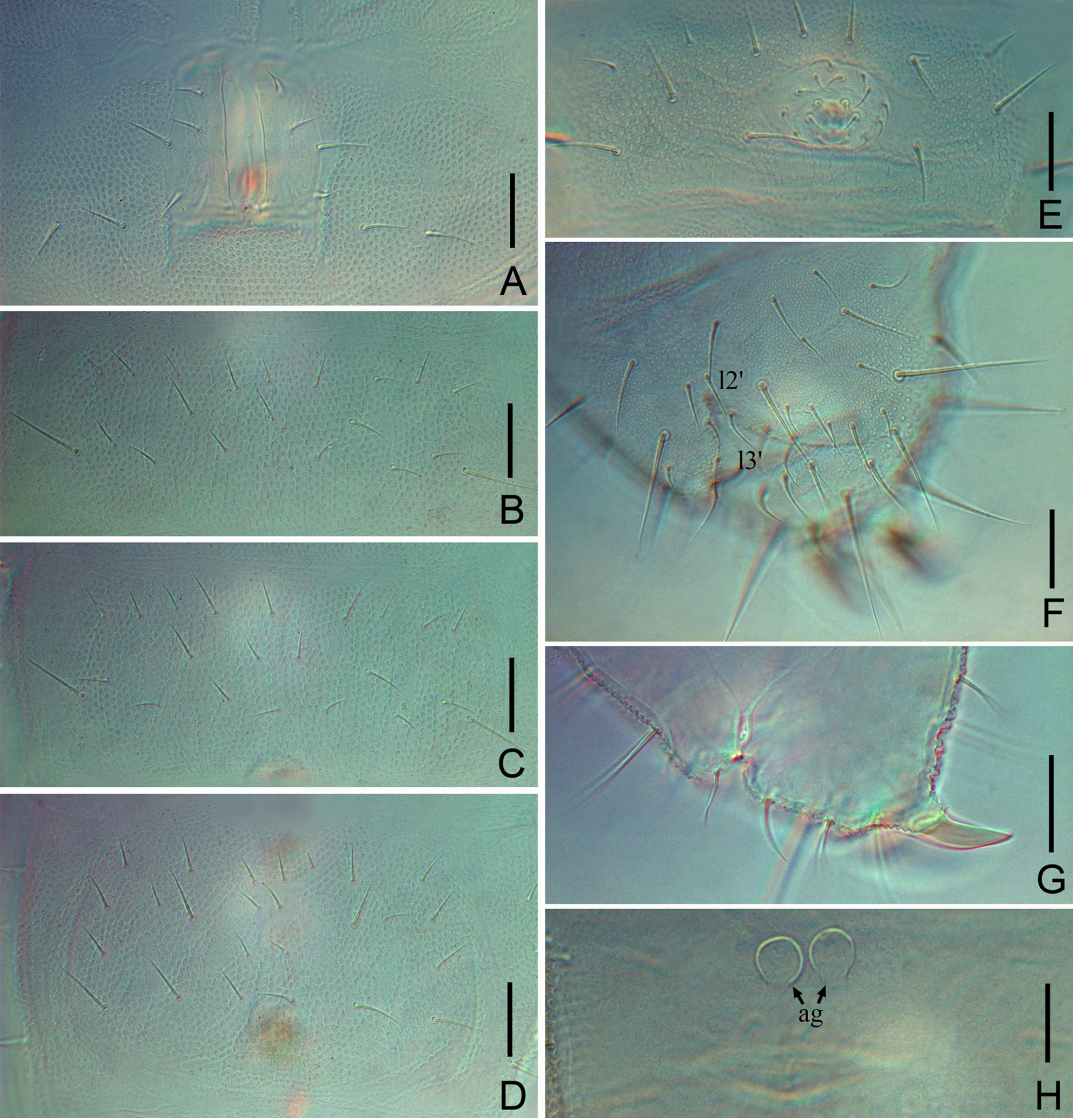
*Metaphorura alticola* sp. nov. **A**. Ventral tube, top view; **B**. Sternite II; **C**. Sternite III; **D**. Sternite IV; **E**. Male genital plate; **F**. Anal lobes; **G**. Anal spine, lateral view; **H**. Sternite V shows accessory glands (ag) and the female genital plate. Scale bars: 20 μm.

Figs 1, 2, 3, 4, 5, Table 2

##### Material examined.

***Holotype***: • female (slide no. XZ-SN-C2024013) (SNHM), China, Xizang, Shannan City, Langkazi County, extracted from soil samples in sparse scrubland close to Yamdrok Lake (Fig. 1A), Alt. 4000 m a.s.l., 29°18'N, 90°38'E, 8-VIII-2024, coll. Y. Bu. ***Paratypes***: • 15 females (slides nos. XZ-SN-C2024010–XZ-SN-C2024012, XZ-SN-C2024014–XZ-SN-C2024018, XZ-SN-C2024020–XZ-SN-C2024026) (SNHM), • 2 males (slides nos. XZ-SN-C2024019, XZ-SN-C2024027) (SNHM), same data as holotype; • 1 female (slides no. XZ-SN-C2024041), China, Xizang, Shannan City, Langkazi County, Pumajiangtang Town, extracted from soil samples in alpine meadow beside lake Pumoyum Co (Fig. 1B), Alt. 5300 m a.s.l., 28°33'N, 90°24'E, 22-VII-2024, coll. Y. Bu. ***Other materials***: • 2 juveniles (slides nos. XZ-SN-C2024028, XZ-SN-C20240292) (SNHM), • 20 adults in alcohol, same data as holotype.

**Table 2.** Dorsal chaetotaxy of *Metaphorura alticola* sp. nov. (holotype).

**Segments**		**Thorax**			**Abdomen**				
		**I**	**II**	**III**	**I**	**II**	**III**	**IV**	**V**
**Dorsal**	**a**	–	10	10	10	10	10	8^3^	10^5^
	**m**	8	8	8	2^1^	4^2^	4^2^	4^2^	–
	**p**	–	8	8	10	10	10	8^4^	8^6^
	**pl**	2	3	3	2	3	3	6	1
**Ventral**		0	2	2	12	20	23	26	16+(6+2)^7^

^1^ seta m4 present; ^2^ seta m3 and m4 present; ^3^ seta a5 absent; ^4^ seta p4 absent; ^5^ seta a1–a3 microseta, a4 as macroseta; ^6^ sensory seta p3 slightly differentiated, seta p4 as microseta; ^7^ female genital plate with 6 circumgenital and one pair of eugenital setae.

##### Description.

**Figure 6.**
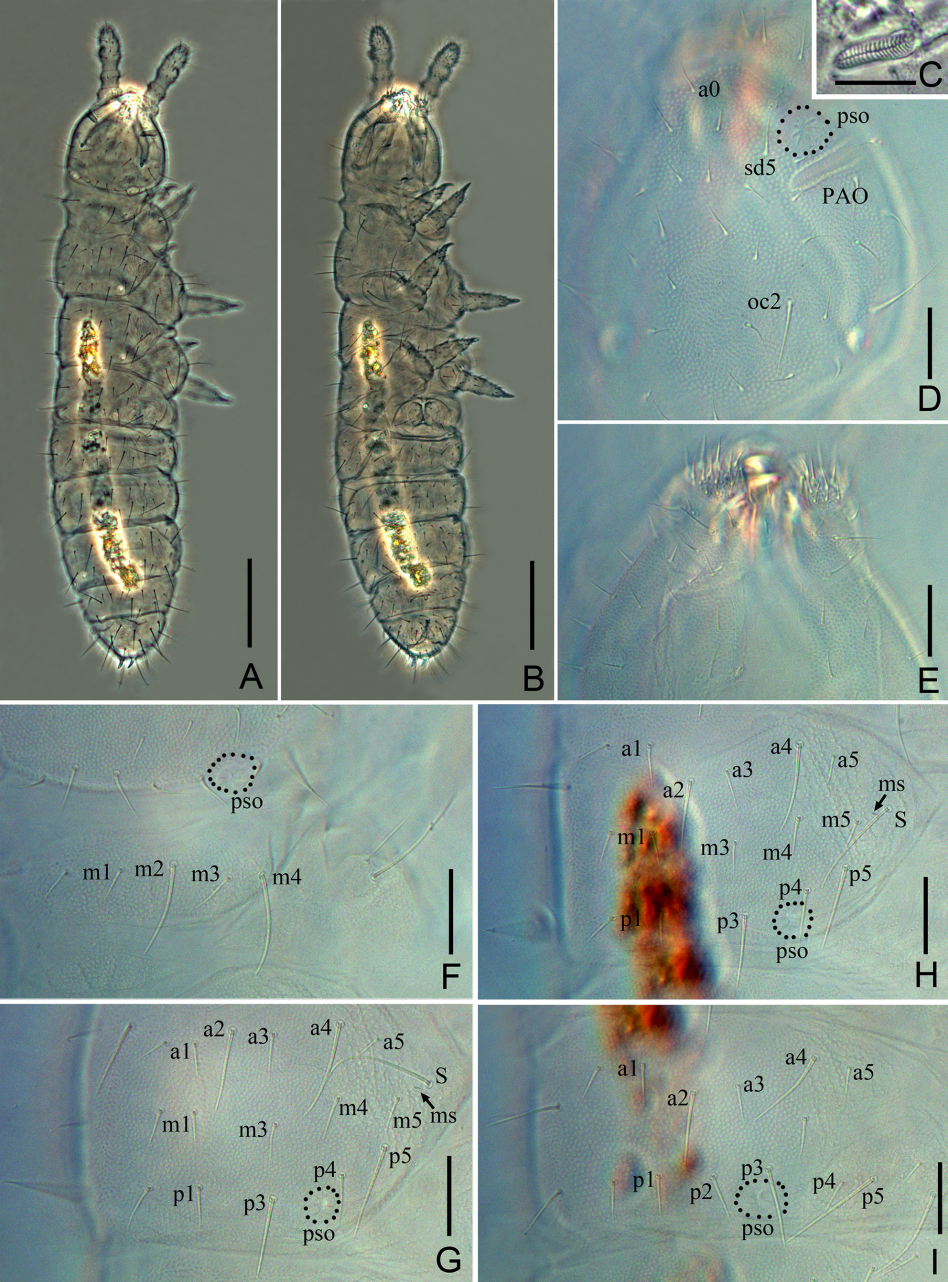
*Mesaphorura macrochaeta* Rusek, 1976. **A**. Habitus, dorsolateral view; **B**. Habitus, ventrolateral view; **C**. Postantennal organ; **D**. Head, dorsal view; **E**. Head, ventral view; **F**. Th. I, right side; **G**. Th. II, right side; **H**. Th. III, right side; **I**. Abd. I, right side. Scale bars: 100 μm (**A, B**); 20 μm (**C–I**).

**Figure 7.**
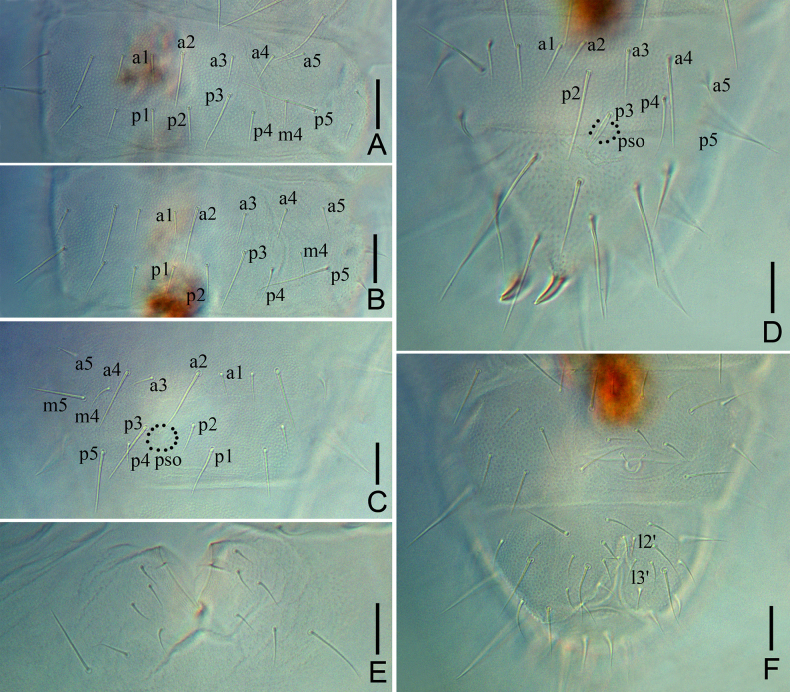
*Mesaphorura macrochaeta* Rusek, 1976. **A**. Abd. II, right side; **B**. Abd. III, right side; **C**. Abd. IV, left side; **D**. Abd. V and VI, right side; **E**. Ventral tube, top view; **F**. Female genital plate and anal lobes. Scale bars: 20 μm.

***Adult body*** robust, white in alcohol (Fig. 1H), 0.93 mm long on average (0.78–1.10 mm, *n* = 19), holotype 1.08 mm (Fig. 2A, B). Setae extremely differentiated into micro- and macrosetae, especially on the head, macrosetae (38–45 μm) about 6–7 times longer than microsetae (5–8 μm). Granulation of integument fine, with stronger granulation only on head and Abd VI (Figs 2, 3, 4). Pseudocellar formula: 11/111/11111. All pseudocelli composed of two rows of parallel stripes in centre (type II, Figs 2D, 2F, 2I, 3C, 4A–G), 6–7 μm in diameter, on Th. I posterior to level of setae m2–m3, close to hind margin (Fig. 2I); on Th. II and III between setae p3/p4, close to p3 (Fig. 4A, B); on Abd. I–III posterior to seta p3 (Fig. 4 C–E); on Abd. IV close to seta p3 (Fig. 4F); on Abd. V on border of Abd. VI (Fig. 4G).

***Head*** seta a0 present (16–18 μm), c1 absent, oc2 as macroseta (35–38 μm), sd5 as mesoseta (18–22 μm) (Fig. 2D, F). Postantennal organ 20–27 μm long, 7–8 μm wide, composed of 12–14 elliptical vesicles arranged in two rows, in a deep furrow (Figs 2C, 3C). Labrum with 4/4/2 setae. Labium with five papillae, six apical guard setae, six proximal setae, four basomedian setae, and five basolateral setae (Fig. 2E). Ventral head with 3+3 axial setae, posterior one (plb3) as macrosetae (Fig. 2G).

***Antenna*** (100–125 μm) shorter than head (130–155 μm). Ant. I and II with 7 and 11 setae, respectively (Fig. 3A, B). Antennal segment IV with five slightly thickened sensilla a–e, sensilla a, c, e long and slightly curved toward inside, b and d slightly shorter (Fig. 3A). Small microsensillum, subapical organite, and one small apical vesicle present (Fig. 3A). Antennal organ III with two small sensory rods between two thick sensory clubs bent toward each other, concealed behind three papillae and four guard setae, and one ventral sensory club (Fig. 3A, B).

***Legs*** without clavate tenant hairs (Fig. 2H). Subcoxa, coxa, trochanter, femur, and tibiotarsus with 0/3/3; 3/7/7; 5/5/4; 8/8/8; 10/10/10 setae on leg I, II and III, respectively (Fig. 2H), tibiotarsi each with 6+4 setae (A1 to A6, B4 to B7, and M absent) (Fig. 3E). Anal lobes with setae 12’ and l3’ (Fig. 5F). Claw 20–23 μm long, untoothed, with short empodial appendage (3–5 μm) (Fig. 3E).

***Adult chaetotaxy*** given in Figs 2I, 2J, 4A–G, and Table 2. Setae on Th. I length as 10–12 μm for m1 and m3, 21–27 μm for m2 and m4 (Fig. 2I). Microsensilla present on Th. II–III, and lateral sensory setae S 27–33 μm long (Fig. 4A, B). Thorax with 0, 2, 2 ventral setae. Abd. I–III each with 2+2 axial setae dorsally, setae m4 present on Abd. I, setae m3 and m4 present on Abd. II–III (Fig. 4C–E). Abd. IV without seta px, setae m3 and m4 present, p1 (10–11 μm) shorter than p2 (22–26 μm) (Fig. 4F). Abd. V with sensory seta p3 (16–23 μm) slightly differentiated; seta a1 (7–10 μm) slightly shorter than a2 and a3 (12–17 μm), a4 as macroseta (33–37 μm), and p4 as microsetae (Fig. 4G). Crescentic ridges on Abd. VI absent. Abd. VI with distinct dorsal secondary granulations, median process between the anal spines not observed (Fig. 4H). Anal spines robust, 20–25 μm in length, width of base about 0.5 of length (Figs 3D, 4H, 5G).

***Ventral tube*** with 4+4 apical setae and 2+2 basal setae (Fig. 5A). Number of ventral setae on Abd. II, III, and IV variable, with 18–20, 19–23, and 25–26 setae, respectively (Fig. 5B–D). Female genital plate with 3 pairs of pregenital setae, 2 or 3 pairs of circumgenital and 1 pair of eugenital setae (Fig. 2J). One pair of accessory glands close to female genital plate observed in fully matured females (Fig. 5H). Male genital plate with 3 pairs of pregenital setae, 17–21 circumgenital setae (Fig. 5E).

##### Etymology.

The species name alticola refers to the extremely high altitude (4000–5300 m above sea level) of the type localities.

##### Distribution.

Known only from Xizang.

##### Diagnosis.

*Metaphorura alticola* sp. nov. is characterised by the well-differentiated setae on the dorsal side of the body, the presence of pseudocelli on thoracic segment I, the few and simple vesicles (12–14) on PAO, the 11/111/11111 pseudocellar formula, all pseudocelli of type II, p4 on abdominal segment V as microsetae, less differentiated sensory setae p3 on abdominal segment V, absence of median process on Abd VI, and the robust anal spines. Bisexual.

##### Remarks.

*Metaphorura alticola* sp. nov. is similar to *Metaphorura motuoensis* in having the same formula of pso on the body, few simple vesicles on PAO, and the absence of the median process on Abd. VI. These species can be distinguished by several subtle differences after careful comparison: the dorsal chaetotaxy (microsetae strongly differentiated from macrosetae in *M. alticola* sp. nov. vs microsetae not strongly differentiated from macrosetae in *M. motuoensis*), number of vesicles on PAO (12–14 in *Metaphorura alticola* sp. nov. vs 14–16 in *M. motuoensis*), and the shape of anal spines on Abd. VI (robust and thick in *Metaphorura alticola* sp. nov. vs slim in *M. motuoensis*). The K2P genetic distance of the COI gene between them is 0.2022 (Suppl. material 1), further supporting our morphological identification. *Metaphorura alticola* sp. nov. can also be easily separated from *Metaphorura zhongi* Bu & Gao, 2017 described from Xizang by the shape of PAO (12–14 irregular vesicles in *Metaphorura alticola* sp. nov. vs 17–18 finger-shaped vesicles in *M. zhongi*), formula of pso on the body (11/111/11111 in the new species vs 11/122/22221 in *M. zhongi*).

The paired globular structure inside the body near the female genital plate was observed in all females of *Metaphorura alticola* sp. nov., which was also observed in European *Metaphorura* species (Dr. Wanda Weiner pers. comm.). After careful comparison, we found that its shape resembles the accessory glands observed in *Orchesella villosa* (Linnaeus, 1767) (Dallai et al. 2008), but a similar structure was not found in *Mesaphorura sylvatica* (Rusek, 1971) (Panina et al. 2019) and *M. macrochaeta* Rusek, 1976 from Xizang. We then named this structure as accessory glands in the present paper.

#### Genus Mesaphorura

Taxon classificationAnimaliaPoduromorphaTullbergiidae

Börner, 1901

0FF49BAF-F269-5269-BF88-C7BB2C7985FA

##### Type species.

*Mesaphorura krausbaueri* Börner, 1901.

#### Mesaphorura macrochaeta

Taxon classificationAnimaliaPoduromorphaTullbergiidae

Rusek, 1976

D7D37A78-171C-56E7-ACC4-C231284EBF13

Figs 6, 7, Table 3

##### Material examined.

China – Xizang • 13 females; Linzhi City, Bayi District, Biri Sacred Mountain; 29°39'N, 94°24'E; 3000 m a.s.l.; coll. Y. Bu; 8-VII-2024; extracted from soil samples in broadleaf forest (Fig. 1C); SNHM slides nos. XZ-LZ-C2024032–XZ-LZ-C2024038 • 18 females; Linzhi City, Gongbujiangda County; 29°59'N, 93°53'E; 3700 m a.s.l.; coll. Y. Bu; 22-IX-2024; extracted from soil samples in bush forest beside the lake Basong Co (Fig. 1D); SNHM slides nos. XZ-LZ-C2024051–XZ-LZ-C2024060.

**Table 3.** Dorsal chaetotaxy of *Mesaphorura macrochaeta* Rusek, 1976.

**Segments**		**Thorax**			**Abdomen**				
		**I**	**II**	**III**	**I**	**II**	**III**	**IV**	**V**
**Dorsal**	**a**	—	10	10	10	10	10	10	10^3^
	**m**	8	8	8	2^1^	2^1^	2^1^	4^2^	—
	**p**	—	8	8	10	10	10	10	8^4^
	**pl**	2	3	3	2	3	3	4	1
**Ventral**		0	2	2	12	20	22	24–26	16+(3+2)^4^

^1^ seta m4 present; ^2^ setae m4 and m5 present; ^3^ seta a1–a3 as microseta, a4 as macroseta; ^4^ sensory seta p3 slightly spindle-like, seta p2 and p5 as macroseta; ^5^ female genital plate with 3 circumgenital and one pair of eugenital setae.

##### Description of Chinese materials.

***Adult body*** 0.63 mm long on average (0.51–0.77 mm, *n* = 31). Dorsal setae well differentiated into micro- and macrosetae (Figs 6, 7). Granulations on body fine. Pso formula as 11/011/10011 from head to abdomen, 8–10 μm in diameter, type I (Figs 6D, 6F–I, 7C). On Th. II and III each with one pair of lateral pso posterior to seta p4 (Fig. 6G, H); pso on Abd. I– IV posterior to seta p3 (Figs 6I, 7A–C), on Abd. V between the borders of Abd. V and VI (Fig. 7D).

***Cephalic seta*** a0 and sd5 as mesosetae, 15–20 μm, c1 absent, oc2 as macroseta, 20–22 μm (Fig. 6D). Postantennal organ 17–22 μm long and 5–7 μm wide, composed of 40–48 elliptical vesicles arranged in two rows (Fig. 6C). Labrum with 4/5/4 setae. Labium with 5 papillae, 6 apical guard setae, 6 proximal setae, 4 basomedian setae, and 5 basolateral setae (Fig. 6E).

***Antenna*** (70–90 μm) shorter than head (98–122 μm) (Fig. 6A, B). Antennal segment IV with 5 thickened sensilla a–e with short heel, sensillum b thicker than others, small microsensillum, subapical organite and 1 apical vesicle present. Antennal organ III consists of 2 small sensory rods concealed behind 1 large papilla and 2 thick sensory clubs bent toward each other, with 4 guard setae; 1 large ventral sensory club present.

***Legs*** short, without clavate tibiotarsal hairs. Coxa, trochanter, femur, and tibiotarsus with 3/7/7; 5/5/4; 9/9/9; 11/11/11 setae on Leg I, II, and III, respectively. Claw 14–18 μm long, with tiny empodial appendage. Anal lobes with setae l3’ and 12’ present (Fig. 7F). Anal spines short, 9–12 μm long (Fig. 7D).

***Adult chaetotaxy*** given in Figs 6F–I, 7A–Dand Table 3. Microsensilla present on Th. II–III, lateral sensilla 31–34 μm long (Fig. 6H, G). Th. II and III with a2 seta present. Thorax with 0, 2, 2 ventral setae. Abd. I–III with 2+2 axial setae each and seta m4 present (Figs 6I, 7A, 7B). Abd. IV with setae m4 and m5 present, p1 as macrosetae (24–28 μm) and in posterior position to mesoseta p2 (13–17 μm), transversal groove present (Fig. 7C). Abdominal segment V with microsetae a1–a3 present, sensillum p3 spindle-like, 12–14 μm in length; a4 (27–33 μm) and p2 (30–33 μm) as macroseta (Fig. 7D). Crescentic ridges on abdominal segment VI present (Fig. 7D).

***Ventral tube*** having 4+4 apical setae and 2+2 basal setae, with 1 pair of basal setae as macrosetae (Fig. 7E). Abd. II, III and IV with 20, 22, and 24–26 ventral setae, respectively. Female genital plate with 6+6 pregenital setae, 3 circumgenital setae, 1 pair of eugenital setae, and 2 pairs of postgenital setae (Fig. 7F).

##### Distribution.

China, Xizang (new record to China); known from Canada, Greenland, arctic Islands (from Spitsbergen to the Northeast Asia Islands), Hawaii, Antarctica, Europe and Asia; probably cosmopolitan (Dunger and Schlitt 2011).

##### Diagnosis.

*Mesaphorura macrochaeta* is characterised by the well-differentiated setae on the dorsal side of the body, PAO with 38–48 elliptical vesicles, the pseudocellar formula 11/011/10011, all pseudocelli of type I, pso on Th. II and III between setae p3/p4, microsetae a1–a3 on present on abd. V, sensory setae p3 on Abd. V spindle-like, anal lobes with both setae l3’ and 12’ present, and Abd. IV with transversal groove present. Parthenogenetic, only females known.

##### Remarks.

*Mesaphorura macrochaeta* was originally described from the Saanich Peninsula, British Columbia, Canada. Later, it was found all over the world, widely distributed in coniferous and broadleaf forests, from mountain to alpine meadows and urban sites, and sometimes also in coastal areas (Rusek 1976; Thibaud and Weiner 1994; Dunger and Schlitt 2011). We found it in the broadleaf forest or bush in Xizang at a high density. The K2P genetic distance of the COI gene between the Chinese specimen and the Danish specimen is only 0.0061 (Suppl. material 1).

#### Mesaphorura hylophila

Taxon classificationAnimaliaPoduromorphaTullbergiidae

Rusek, 1982

9C5C395A-812D-52EB-A9E4-95F2267801D6

##### Material examined.

China – Xizang • 20 females; Lhasa City, Chengguan District, Xizang Academy of Agricultural and Animal Husbandry Sciences; 29°38'N, 91°02'E; 3650 m a.s.l.; coll. Y. Bu; 29-VI-2024; extracted from soil samples collected under the root of the poplar tree in the campus (Fig. 1G); SNHM slides nos. XZ-SN-C2024006–XZ-SN-C2024009 • 6 females; Naqu City, Jiali County; 30°32'N, 94°06'E; 3368 m a.s.l.; coll. Y. Bu; 18-XII-2024; extracted from soil samples from coniferous forest (Fig. 1F); SNHM slides nos. XZ-NQ-C2024064, XZ-NQ-C2024065.

##### Distribution.

China (Hebei, Xizang) (Bu and Gao 2017a, 2017b); widely distributed in the Palaearctic (Norway, Greenland, Scandinavia, Spain, Poland, Italy, Slovakia, Germany, Russia) (Dunger and Schlitt 2011).

##### Remarks.

*Mesaphorura hylophila* has been recorded in Linzhi City, Xizang (Bu and Gao 2017b) and for the first time found in Lhasa and Naqu Cities.

#### Mesaphorura krausbaueri

Taxon classificationAnimaliaPoduromorphaTullbergiidae

(Börner, 1901)

31C7B447-B868-5FDA-B4CA-CB90F6EB5A09

##### Material examined.

China – Xizang • 1 female; Linzhi City, Gongbujiangda County; 29°59'N, 93°53'E; 3700 m a.s.l.; coll. Y. Bu; 22-IX-2024; extracted from soil samples in a bush forest beside the lake Basong Co (Fig. 1D); SNHM slide no. XZ-LZ-C2024060.

##### Distribution.

China (Shanghai, Sichuan, Xizang–new record); widely distributed in Norway, Greenland, Scandinavia, Spain, Poland, Italy, Slovakia, Germany, Russia, and America (Dunger and Schlitt 2011).

##### Remarks.

*Mesaphorura krausbaueri* was originally described from Germany and for the first time found in Xizang. The main difference to *M. macrochaeta* is the absence of seta 12’ on anal lobes. The specimen from Xizang has relatively less differentiated setae on the dorsal side of the body.

#### Mesaphorura pacifica

Taxon classificationAnimaliaPoduromorphaTullbergiidae

Rusek, 1976

94A59BD6-9695-541B-ACEA-67C754E34767

##### Material examined.

China – Xizang • 12 females; Shannan City, Langkazi County, Pumajiangtang Town; 28°33'N, 90°24'E; 5300 m a.s.l.; coll. Y. Bu; 22-VII-2024; extracted from soil samples in alpine meadow beside lake Pumoyum Co (Fig. 1B); SNHM slides nos. XZ-SN-C2024042–XZ-SN-C2024044 • 1 female; Naqu City, Bange County; 31°50'N, 89°37'E; 4552 m a.s.l.; coll. Y. Bu; 17-IX-2024; extracted from soil samples in the grassland (Fig. 1E); SNHM slide no. XZ-NQ-C2024048.

##### Distribution.

China (Hebei, Shanghai, Xinjiang, Xizang–new record) (Bu and Gao 2017a, 2017b; Gao and Bu 2022); North America; North Africa; Iraq (Dunger and Schlitt 2011).

##### Remarks.

*Mesaphorura pacifica* is widely distributed in the Palaearctic and nearctic region. In China, the species has been recorded in Hebei and Xinjiang (Bu and Gao 2017a; Gao and Bu 2022) and for the first time in Xizang in this paper.

#### Mesaphorura yosii

Taxon classificationAnimaliaPoduromorphaTullbergiidae

(Rusek, 1967)

DA8BE98B-581F-5D71-9B35-77B37C2F3E06

##### Material examined.

China – Xizang • 5 females; Linzhi City, Bayi District, Biri Sacred Mountain; 29°39'N, 94°24'E; 3000 m a.s.l.; coll. Y. Bu; 8-VII-2024; extracted from soil samples in broad-leaf forest (Fig. 1C); SNHM slide no. XZ-LZ-C2024039.

##### Distribution.

Cosmopolitan. In China, the species was found in Fujian, Guangdong, Hainan, Hebei, Hunan, Jiangsu, Shandong, Shanghai, Sichuan, Xinjiang, Xizang, Yunnan and Zhejiang (Gao and Bu 2022).

##### Remarks.

*Mesaphorura yosii* was previously recorded in Sejila Mountain, Motuo County, Bomi County, and Lulang forest farm of Linzhi City (Bu and Gao 2017b) and for the first time in Biri Sacred Mountain. This species is probably widely distributed in the Southeast area of Xizang.

### Genetic divergence analysis

**Figure 8.**
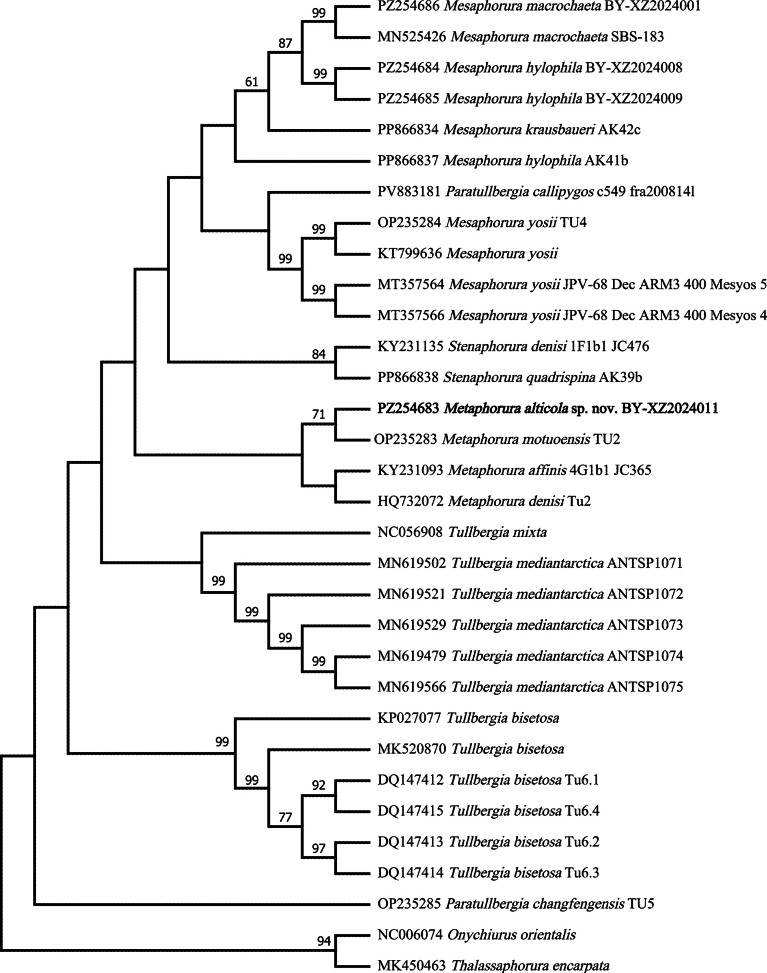
Neighbour-joining tree (Jukes–Cantor model, bootstrap 1000 replicates) of Tullbergiidae inferred from COI gene sequences. Numbers at the nodes show the bootstrap values > 50%.

Four DNA barcodes (COI gene) of *Metaphorura alticola* sp. nov., *M. macrochaeta*, and *M. hylophila* from Xizang were newly sequenced and registered in GenBank (Table 1), each of them composed of 658 base pairs. The pairwise genetic distance of 30 sequences of Tullbergiidae species based on the K2P model is given in the Suppl. material 1. The genetic distances of the COI gene among different taxonomic levels of Tullbergiidae are analysed and given in Table 4. Based on COI gene sequences, a neighbour-joining tree was constructed for 15 species of Tullbergiidae, which is the first comprehensive gene tree for the family with most taxa included (Fig. 8).

**Table 4.** Genetic distances at different taxonomic levels of Tullbergiidae analysed by mitochondrial COI gene (K2P model).

**Level**	**Mean**	**Minimum**	**Maximum**
***Metaphorura alticola* sp. nov**. vs congeners	0.2454	0.2022	0.2778
Interspecific distances within the genus *Metaphorura*	0.2402	0.2022	0.2778
Interspecific distances within the genus *Mesaphorura*	0.2540	0.1976	0.3094
Conspecific distances of *Mesaphorura macrochaeta*	0.0061	0.0061	0.0061
Conspecific distances of *Mesaphorura hylophila*	0.0904	0.0000	0.2855
Conspecific distances of *Mesaphorura yosii*	0.0852	0.0077	0.1270
Interspecific distances within the genus *Paratullbergia*	0.2608	0.2608	0.2608
Interspecific distances within the genus *Stenaphorura*	0.2118	0.2118	0.2118
Interspecific distances within the genus *Tullbergia*	0.2725	0.2835	0.2567
Conspecific distances of the genus *Tullbergia bisetosa*	0.0076	0.0000	0.0223
Conspecific distances of the genus *Tullbergia mediantarctica*	0.0000	0.0000	0.0000
Intergeneric distances between five genera	0.2653	0.2185	0.3179
Tullbergiidae species vs Onychiuridae outgroups	0.2804	0.2325	0.3331

## Discussion

**Figure 9.**
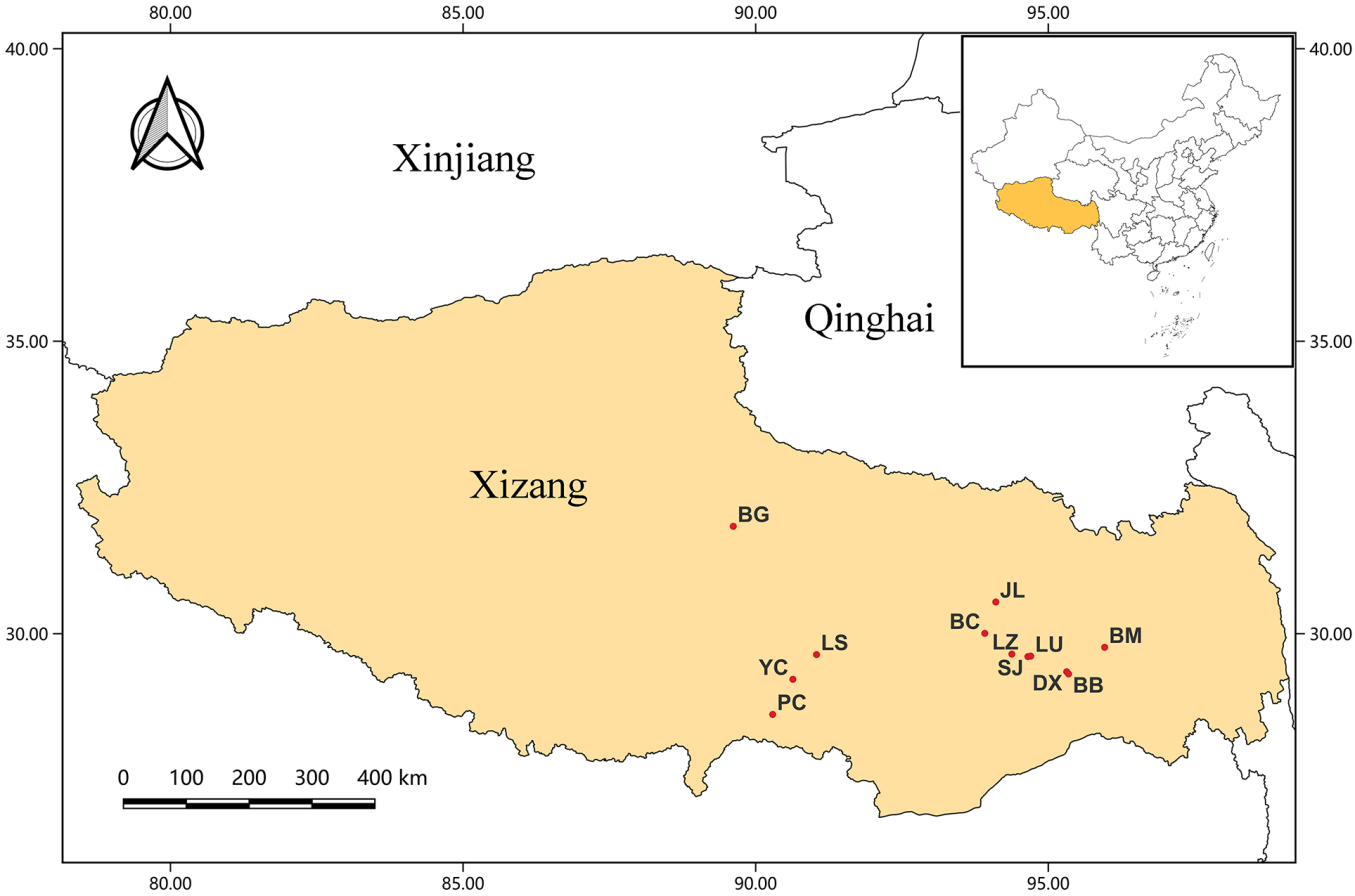
Distribution records of Tullbergiidae in Xizang. BB – Beibeng Town, Motuo County, Linzhi City; BC – Lake Basong Co, Gongbujiangda County, Linzhi City; BG – Bange County, Naqu City; BM – Songzong town, Bomi County; DX – Dexing Town, Motuo County, Linzhi City; JL – Jiali County, Naqu City; LS – Chengguan District, Lhasa City; LU – Lulang forest farm, Linzhi City; LZ – Bayi District, Linzhi City; PC – Lake Pumoyum Co, Langkazi County, Shannan City; SJ – Sejila Mountain, Linzhi City; YC – The Yamdrok Lake, Langkazi County, Shannan City.

*Metaphorura alticola* sp. nov. is the third Chinese species of the genus described from Xizang but was found at an extremely high altitude. In contrast, the other two species, *M. motuoensis* and *M. zhongi*, are known to occur at relatively lower altitudes (Bu and Gao 2017b, 2019). Until now, nine species of Tullbergiidae belonging to three genera have been recorded from 12 sites in Xizang (Fig. 9), and their distributions are listed in Table 5. Among them, three species of the genus *Metaphorura* are only known in Xizang at present, while five species of *Mesaphorura* are widely distributed species, and a single species of the genus *Prabhergia* has also been recorded in Yunnan Province, Southwest China.

**Table 5.** Distribution of species of Tullbergiidae in Xizang.

**Species**	**Sites**											
	**BB**	**BC**	**BG**	**BM**	**DX**	**JL**	**LS**	**LU**	**LZ**	**PC**	**SJ**	**YC**
*Metaphorura alticola***sp. nov**.										+		+
*Metaphorura motuoensis*					+						+	
*Metaphorura zhongi*											+	
*Mesaphorura macrochaeta*		+							+			
*Mesaphorura hylophila*				+		+	+					
*Mesaphorura krausbaueri*		+										
*Mesaphorura yosii*	+			+	+			+	+		+	
*Mesaphorura pacifica*			+							+		
*Prabhergia imadatei*					+							

Notes: the abbreviations for the sites are the same as used in Fig. 9; “+” symbol means “present”.

### Key to the Tullbergiidae species from the Xizang Autonomous Region, China

The nine species of Tullbergiidae recorded from Xizang can be separated by the following key.

**Table:** 

1	PAO with 7 irregular, large vesicles arranged in two rows	***Prabhergia imadatei* Tamura & Zhao, 1996**
–	PAO with more than 12 elongate vesicles arranged in 2 rows	**2**
2	Ant. III with 3 protecting papillae; PAO with fewer vesicles	**3**
–	Ant. III with 1 large protecting papillae; PAO with numerous vesicles	**5**
3	Formula of pso on the body as 11/122/22221	***Metaphorura zhongi* Bu & Gao, 2019**
–	Formula of pso on the body as 11/111/11111	**4**
4	Microsetae strongly differentiated from macrosetae; anal spines on Abd. VI thick; PAO with 12–14 vesicles	***Metaphorura alticola* sp. nov**.
–	Microsetae strongly differentiated from macrosetae; anal spines on Abd. VI slim; PAO with 14–16 vesicles	***Metaphorura motuoensis* Bu & Gao, 2017**
5	Abd. V with 5 pairs of a-row setae; a2 seta absent	**6**
–	Abd. V with 4 pairs of a-row setae; a2 seta present	***Mesaphorura hylophila* Rusek, 1982**
6	On Abd. V, seta a2 as mesoseta; sensillum p3 slender	***Mesaphorura pacifica* Rusek, 1976**
–	On Abd. V, seta a2 as microseta; sensillum p3 spindle-like	**7**
7	Seta 12’ on anal lobe absent	***Mesaphorura krausbaueri* (Börner, 1901)**
–	Seta 12’ on anal lobe present	**8**
8	Microsetae distinctly differentiated from macrosetae; Abd. IV with transversal groove; PAO with 38–48 elliptical vesicles arranged in 2 rows	***Mesaphorura macrochaeta* Rusek, 1976**
–	Microsetae less differentiated from macrosetae; Abd. IV without transversal groove; PAO with 36–38 elliptical vesicles arranged in 2 rows	***Mesaphorura yosii* (Rusek, 1967)**

Although *Metaphorura alticola* sp. nov. is very similar to *M. motuoensis* in morphology, the genetic divergence between them is fairly high (0.2022), and the genetic distances between *Metaphorura alticola* sp. nov. and other congeners is 0.2454 on average (0.2022–0.2778), which gives powerful support for our morphological identification. The neighbour-joining tree also indicates that *Metaphorura alticola* sp. nov. is unique but clustered with *Metaphorura motuoensis* (Fig. 8).

For the genetic divergence of different levels, the intergeneric distance between five genera is 0.2653 on average, and the interspecific distances of the same genus are: 0.2402 for *Metaphorura*, 0.2540 for *Mesaphorura*, 0.2608 for *Paratullbergia*, 0.2118 for *Stenaphorura*, and 0.2775 for *Tullbergia* (Table 4). The conspecific distances of *Mesaphorura macrochaeta* between specimens from Xizang and Denmark are only 0.0061. While the two sequences of *Mesaphorura hylophila* from Xizang are identical, they have a fairly large genetic distance with the sequence from Germany (0.2855), indicating that the identification of the species needs further confirmation. For *Mesaphorura yosii*, the distance between two Chinese populations is 0.0077, lower than the distance between two individuals from Panama (0.0145), and the distance between the populations of China and Panama is 0.1223 on average. In contrast, the conspecific distances for the two species of the genus *Tullbergia* are much lower, 0.0000–0.0076 on average. Tullbergiidae species have the highest genetic distances compared with Onychiuridae outgroups, with a value of 0.2804 on average.

The neighbour-joining tree relatively well separated each species in clusters (Fig. 8). Four *Metaphorura* species clustered together, and *Metaphorura alticola* sp. nov. forms a sister group with *M. motuoensis*. All *Mesaphorura* species also clustered together, with *M. macrochaeta*, *M. hylophila*, and *M. krausbaueri* most closely related. However, two species of the genus *Paratullbergia* are separated on the tree: *P. changfengensis* from China forms a single clade basal to all other species, while *P. callipygos* from France clustered with *Mesaphorura* species. Due to the limits of molecular data available and species included, we cannot draw too many conclusions now. A future study with a broader sampling and more molecular markers might clarify the relationships of Tullbergiidae at different taxonomic levels.

## Supplementary Material

XML Treatment for Metaphorura

XML Treatment for Metaphorura alticola

XML Treatment for Mesaphorura

XML Treatment for Mesaphorura macrochaeta

XML Treatment for Mesaphorura hylophila

XML Treatment for Mesaphorura krausbaueri

XML Treatment for Mesaphorura pacifica

XML Treatment for Mesaphorura yosii

## Additional information

### Artificial Intelligence (AI) use

The authors accept full responsibility for the content of the manuscript, including the disclosure of any use of AI.

No AI tools were used in the preparation of this manuscript.

### Author ORCIDs

Y. Bu https://orcid.org/0000-0002-7177-9686

Y. Gao https://orcid.org/0000-0003-0305-3696

### Data availability

All of the data that support the findings of this study are available in the main text or Supplementary Information.

## Supplementary materials

Supplementary material 1 Pairwise distances Data type xls Explanation note **table SS1**. Pairwise distances (K2P model) between COI DNA sequences of Tullbergiidae (distances between the new species and other Tullbergiidae species given in bold, intraspecific distances given in red, other interspecific distances are in regular letters). https://binary.pensoft.net/file/1723929This dataset is made available under the Open Database License (http://opendatacommons.org/licenses/odbl/1.0/). The Open Database License (ODbL) is a license agreement intended to allow users to freely share, modify, and use this Dataset while maintaining this same freedom for others, provided that the original source and author(s) are credited. Yun Bu, Yan Gao

## References

[B1] Bagnall RS (1935) On the classification of the Onychiuridae (Collembola), with particular reference to the Genus *Tullbergia* Lubbock and its Allies. Annals & Magazine of Natural History (Series 10) 15(86): 236–242. 10.1080/00222933508654961

[B2] Bagnall RS (1936) The British Tullbergiinae. Part II. Entomologists Monthly Magazine 72: 34–40.

[B3] Basset Y, Palacios-Vargas JG, Donoso DA, Castaño-Meneses G, Decaëns T, Lamarre GP, De León LF, Rivera M, García-Gómez A, Perez F, Bobadilla R, Lopez Y, Ramirez JA, Montejo Cruz M, Arango Galván A, Mejía-Recamier BE, Barrio H (2020) Enemy-free space and the distribution of ants, springtails and termites in the soil of one tropical rainforest. European Journal of Soil Biology 99: 1–9. 10.1016/j.ejsobi.2020.103193

[B4] Bellinger PF, Christiansen KA, Janssens F (1996–2024) Checklist of the Collembola of the World. http://www.collembola.org [accessed 20 April 2026]

[B5] Börner C (1901) Zur Kenntnis der Apterygoten-Fauna von Bremen und der Nachbardistrikte. Beitrag zu einer Apterygoten-Fauna Mitteleuropas. Abhandlungen des Naturwissenschaftlichen Vereins zu Bremen 17(1): 1–141. 10.5962/bhl.part.18332

[B6] Börner C (1903) Das Genus *Tullbergia* Lubbock. Zoogischer Anzeiger 16: 123–131. 10.5281/zenodo.13442548

[B7] Bu Y, Gao Y (2017a) Two newly recorded species of *Mesaphorura* (Collembola: Tullbergiidae) from China. Entomotaxonomia 39(3): 169–175.

[B8] Bu Y, Gao Y (2017b) Study on Tullbergiidae of Tibet, China I. *Metaphorura*, *Mesaphorura* and *Prabhergia* (Hexapoda, Collembola). ZooKeys 686: 85–94. 10.3897/zookeys.686.11468 PMC5672563 29200916

[B9] Bu Y, Gao Y (2019) Study on Tullbergiidae of Tibet, China II. *Metaphorura zhongi* sp. n. from Sejila Mountain (Hexapoda, Collembola). Species 20: 84–89.

[B10] Cook CE, Yue Q, Akam M (2005) Mitochondrial genomes suggest that hexapods and crustaceans are mutually paraphyletic. Proceedings of the Royal Society B 272(1569): 1295–1304. 10.1098/rspb.2004.3042 PMC1564108 16024395

[B11] Cucini C, Fanciulli PP, Frati F, Convey P, Nardi F, Carapelli A (2021) Re-evaluating the internal phylogenetic relationships of Collembola by means of mitogenome data. Genes 12: 44. 10.3390/genes12010044 PMC7824276 33396901

[B12] Dallai R, Zizzari ZV, Fanciulli PP (2008) Fine structure of the spermatheca and of the accessory glands in *Orchesella villosa* (Collembola, Hexapoda). Journal of Morphology 269: 464–478. 10.1002/jmor.10595 18157861

[B13] Dunger W, Schlitt B (2011) Synopses on Palaearctic Collembola: Tullbergiidae. Soil Organisms 83(1): 1–168.

[B14] Fjellberg A (1991) Tibiotarsal chaetotaxy in Tullbergiinae (Collembola: Onychiuridae). Entomologica Scandinavica 21: 431–434. 10.1163/187631290x00337

[B15] Folmer O, Black M, Hoeh W, Lutz R, Vrijenhoek R (1994) DNA primers for amplification of mitochondrial cytochrome c oxidase subunit I from diverse metazoan invertebrates. Molecular Marine Biology and Biotechnology 3(5): 294–299. 7881515

[B16] Gao Y, Bu Y (2022) New species and records of Tullbergiidae (Collembola, Poduromorpha) from Xinjiang province, Northwest China. Zootaxa 5092(5): 531–544. 10.11646/zootaxa.5092.5.2 35390827

[B17] Greenslade P, Stevens MI, Torricelli G, D’Haese CA (2011) An ancient Antarctic endemic genus restored: morphological and molecular support for *Gomphiocephalus hodgsoni* (Collembola: Hypogastruridae). Systematic Entomology 36(2): 223–240. 10.1111/j.1365-3113.2010.00553.x

[B18] Hall TA (1999) BioEdit: a user-friendly biological sequence alignment editor and analysis program for Windows 95/98/NT. Nucleic Acids Symposium Series 41: 95–98.

[B19] Jagatap H, Monsanto DM, Jansen van Vuuren B, Janion-Scheepers C, Sekar S, Teske PR, Emami-Khoyi A (2019) The complete mitogenome of the springtail *Tullbergia bisetosa*: a subterranean springtail from the sub-Antarctic region. Mitochondrial DNA B Resources 4(1): 1594–1596. 10.1080/23802359.2019.1601514

[B20] Jukes TH, Cantor CR (1969) Evolution of protein molecules. In: Munro HN (Ed.) Mammalian Protein Metabolism. Academic Press, New York, 21–132. 10.1016/b978-1-4832-3211-9.50009-7

[B21] Kimura M (1980) A simple method for estimating evolutionary rate of base substitutions through comparative studies of nucleotide sequences. Journal of Molecular Evolution 16(2): 111–120. 10.1007/bf01731581 7463489

[B22] Kumar S, Stecher G, Li M, Knyaz C, Tamura K (2018) MEGA X: Molecular Evolutionary Genetics Analysis across computing platforms. Molecular Biology and Evolution 35(6): 1547–1549. 10.1093/molbev/msy096 PMC5967553 29722887

[B23] Li JY, Godeiro NN, Gao Y, Bu Y, Li K (2022) New Tullbergiidae mitogenomes as the evidence of sister relationship with Onychiuridae (Hexapoda, Collembola). Zootaxa 5222(5): 467–477. 10.11646/zootaxa.5222.5.5 37044510

[B24] Myburgh M, Chown SL, Daniels SR, Jansen van Vuuren B (2007) Population structure, propagule pressure, and conservation biogeography in the sub-Antarctic: lessons from indigenous and invasive springtails. Diversity and Distributions 13(2): 143–154. 10.1111/j.1472-4642.2007.00319.x

[B25] Panina IV, Potapov MB, Polilov AA (2019) Effects of miniaturization in the anatomy of the minute springtail *Mesaphorura sylvatica* (Hexapoda: Collembola: Tullbergiidae). PeerJ 7: e8037. 10.7717/peerj.8037 PMC6858819 31741793

[B26] Rusek J (1967) Beitrag zur Kenntnis der Collembola (Apterygota) Chinas. Acta Entomologica Bohemoslovaca 64: 184–194.

[B27] Rusek J (1971) Zur Taxonomie der Tullbergia (Mesaphorura) krausbaueri (Börner) und ihrer Verwandten (Collembola). Acta Entomologica Bohemoslovaca 68: 188–206.

[B28] Rusek J (1976) New Onychiuridae (Collembola) from Vancouver Island. Canadian Journal of Zoology 54: 19–41. 10.1139/z76-003

[B29] Rusek J (1982) European *Mesaphorura* species of the sylvatica-group (Collembola, Onychiuridae, Tullbergiinae). Acta Entomologica Bohemoslovaca 79: 14–30.

[B30] Stach J (1954) The Apterygotan Fauna of Poland in Relation to the World-Fauna of this Group of Insects. Family: Onychiuridae. Polska Akademia Nauk, PWN, Kraków, 219 pp.

[B31] Stevens MI, D’Haese CA (2025) Fantastic beasts: ‘giant’ springtails (Collembola) highlight convergent evolution and major changes in the superfamily Neanuroidea. Zoological Journal of the Linnean Society 205(4): zlaf182. 10.1093/zoolinnean/zlaf182

[B32] Tamura H, Zhao L (1996) Two species of the subfamily Tullbergiinae in Xishuangbanna, Southwest China (Collembola: Onychiuridae). Japanese Journal of Entomology 64(4): 790–794.

[B33] Thibaud J-M, Weiner WM (1994) *Psammophorura gedanica* g. n., sp. n. et autres Collemboles interstitiels terrestres de Pologne. Polskie Pismo Entomologiczne 63: 3–15.

[B34] Weiner WM, Najt J (1991) CollembolaPoduromorpha de Nouvelle-Calédonie. 6. OnychiuridaeTullbergiinae. In: Chazeau J, Tillier S (Eds) Zoologia Neocaledonica, Volume 2. Mémoires du Muséum National d’Histoire Naturelle (A) 149: 119–130.

